# Exploring the Role of Artificial Intelligence in Smart Healthcare: A Capability and Function-Oriented Review

**DOI:** 10.3390/healthcare13141642

**Published:** 2025-07-08

**Authors:** Syed Raza Abbas, Huiseung Seol, Zeeshan Abbas, Seung Won Lee

**Affiliations:** 1 Department of Precision Medicine, School of Medicine, Sungkyunkwan University, Suwon 16419, Republic of Korea; shahbaz5.12@skku.edu; 2 Department of Metabiohealth, Sungkyunkwan University, Suwon 16419, Republic of Korea; 825seol@gmail.com; 3 Department of Artificial Intelligence, Sungkyunkwan University, Suwon 16419, Republic of Korea; 4 Personalized Cancer Immunotherapy Research Center, School of Medicine, Sungkyunkwan University, Suwon 16419, Republic of Korea; 5 Department of Family Medicine, Kangbuk Samsung Hospital, School of Medicine, Sungkyunkwan University, 29 Saemunan-ro, Jongno-gu, Seoul 03181, Republic of Korea

**Keywords:** AI in healthcare, smart healthcare, AI capabilities, AI functionalities, narrow AI, Theory of Mind, medical decision support

## Abstract

Artificial Intelligence (AI) is transforming smart healthcare by enhancing diagnostic precision, automating clinical workflows, and enabling personalized treatment strategies. This review explores the current landscape of AI in healthcare from two key perspectives: capability types (e.g., Narrow AI and AGI) and functional architectures (e.g., Limited Memory and Theory of Mind). Based on capabilities, most AI systems today are categorized as Narrow AI, performing specific tasks such as medical image analysis and risk prediction with high accuracy. More advanced forms like General Artificial Intelligence (AGI) and Superintelligent AI remain theoretical but hold transformative potential. From a functional standpoint, Limited Memory AI dominates clinical applications by learning from historical patient data to inform decision-making. Reactive systems are used in rule-based alerts, while Theory of Mind (ToM) and Self-Aware AI remain conceptual stages for future development. This dual perspective provides a comprehensive framework to assess the maturity, impact, and future direction of AI in healthcare. It also highlights the need for ethical design, transparency, and regulation as AI systems grow more complex and autonomous, by incorporating cross-domain AI insights. Moreover, we evaluate the viability of developing AGI in regionally specific legal and regulatory frameworks, using South Korea as a case study to emphasize the limitations imposed by infrastructural preparedness and medical data governance regulations.

## 1. Introduction

Artificial Intelligence (AI) is playing an increasingly pivotal role in advancing smart healthcare systems by enhancing diagnostic accuracy, enabling personalized treatment, and improving operational efficiency. For instance, AI models have shown great promise in biomedical signal processing as seen in anesthesia stage classification using near-infrared spectroscopy signals [1] and in the enhancement of spin-exchange relaxation-free magnetometers for better physiological sensing [2]. Moreover, sensor calibration improvements like in situ magnetic field compensation for magnetometers [3] and neuromorphic-enabled cell sorting [4] further exemplify the integration of AI into medical instrumentation. In the realm of predictive diagnostics, machine learning approaches are being leveraged for the early detection of conditions such as sepsis [5] and perioperative neurocognitive disorders [6], while outlier detection models contribute to identifying abnormal clinical data patterns [7]. Deep learning also supports precise evaluation in diseases like ulcerative colitis through lesion-level analysis [8] and enhances post-treatment outcomes as demonstrated in AI-assisted nutritional management for cancer patients [9].

The integration of AI into healthcare systems has led to a new era of smart healthcare, characterized by increased diagnostic precision, personalized treatment recommendations, and streamlined clinical workflows [10,11]. Smart healthcare refers to the use of advanced technologies, particularly AI, to improve the quality, accessibility, and efficiency of healthcare services while supporting clinicians and empowering patients. AI in this context acts as a transformative force augmenting human intelligence, automating labor intensive processes, and enabling data-driven clinical decisions [12].

Beyond clinical applications, AI contributes significantly to molecular biology, mental health, and healthcare systems management. In genomics, models like GenoM7GNet [13] and integrative deep learning approaches for RNA structure prediction [14] are advancing our understanding of molecular mechanisms. Mental health research benefits from AI-enabled multimodal analysis, helping elucidate the pathways involved in schizophrenia [15] and postpartum depression [16]. Furthermore, the optimization of pharmaceutical analysis using AI-enhanced HPLC-MS/MS workflows [17] and the deployment of AI-driven control systems in teleoperation [18] illustrate its broader role in healthcare operations. Spatial analytics have also been applied to map the medical device industry in China, aiding strategic health system planning [19]. Collectively, these developments underscore the multifaceted capabilities of AI in transforming healthcare from molecular diagnostics to large-scale public health and industrial applications.

Over the past decade, the rapid digitization of medical records, wearable technologies, and diagnostic imaging has generated vast amounts of healthcare data. This explosion of data, coupled with advancements in machine learning (ML) algorithms, has significantly accelerated the adoption of AI in clinical environments [20]. As healthcare systems worldwide grapple with increasing demand, aging populations, and chronic disease burdens, AI offers scalable solutions that can deliver more efficient and equitable care [21,22].

The current applications of AI in smart healthcare are predominantly classified as Narrow AI systems, task-specific models developed to perform clearly defined functions such as interpreting medical images, detecting anomalies in physiological signals, triaging patients, or assisting in robotic surgeries [23]. While these systems lack the general reasoning capabilities inherent to humans, they can often surpass clinician performance in specialized domains when trained on high-quality datasets. For instance, deep learning (DL) models have demonstrated expert-level accuracy in diagnosing conditions like pneumonia, diabetic retinopathy, cardiovascular diseases, and breast cancer from medical imaging data [24,25]. Beyond diagnostics, AI-based virtual assistants such as Wysa and Woebot are proving effective in supporting mental health by delivering around the clock cognitive behavioral therapy and emotional support [26]. However, despite these advancements, the integration of emerging AI techniques such as federated learning remains in its early stages particularly in South Korea, where pilot projects face substantial interoperability challenges due to the heterogeneity of hospital IT infrastructures [27].

**Figure 1:**
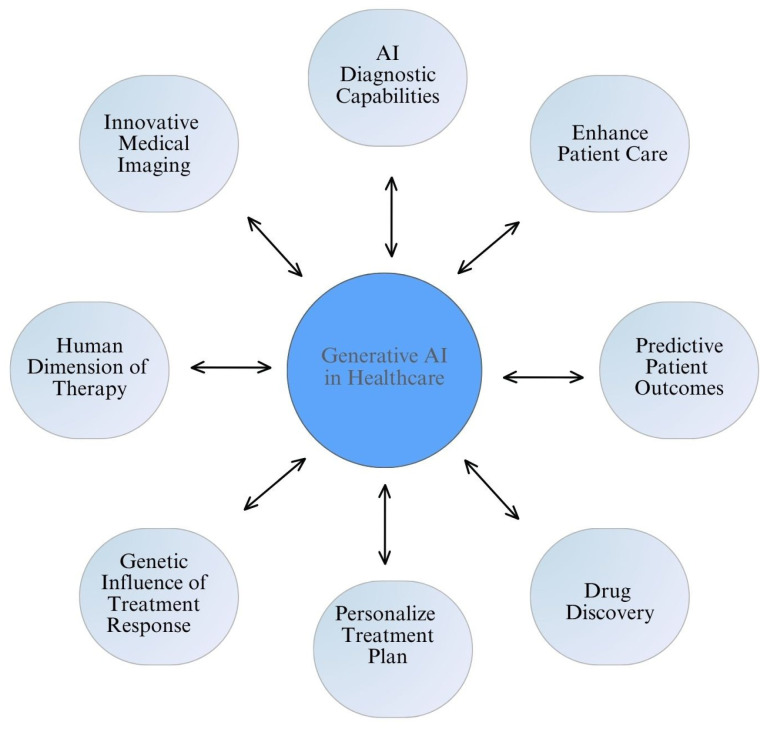
Key applications and benefits of generative AI in healthcare. This schematic illustrates the multifaceted contributions of generative AI, including enhanced diagnostic capabilities, predictive patient outcomes, personalized treatment plans, drug discovery, and support for the human dimension of therapy. These interconnected functions highlight the potential of generative AI to transform both clinical practice and biomedical research.

However, although Narrow AI is very useful, it can only perform specific tasks and cannot adapt to different types of problems. Because of this limitation, researchers are now focusing more on developing AGI, which aims to think and understand like humans, including reasoning, abstract thinking, and recognizing emotions [28]. Although AGI is still mostly a theoretical concept, it has great potential in healthcare. An AGI system could combine different types of patient information, like doctor’s notes, lab tests, genetic data, and medical images, to provide complete and personalized care advice. However, because of the current limitations in technology, as well as ethical and safety concerns, these systems are not yet used in real medical practice. AGI in healthcare builds upon the capabilities of generative AI by enabling more advanced and adaptive applications such as multimodal patient understanding, where diverse data types like clinical notes, imaging, and genomic data are seamlessly integrated for deeper insights. It supports real-time clinical decision-making by offering dynamic, context-aware recommendations during diagnosis and treatment. These advancements make healthcare more personalized, adaptive, and empathetic [29,30], expanding the transformative potential shown in Figure 1.

In addition to capability-based classification, AI in smart healthcare can be understood through its functional architecture, which defines how the system operates. The simplest functional class, reactive machines, responds only to current inputs and lacks memory of past interactions. These systems have been used in early rule-based alert systems and decision trees but are increasingly being replaced by limited memory systems. Limited Memory AI can analyze historical data to improve predictive accuracy and is widely used in patient monitoring, chronic disease management, and personalized treatment planning [31].

Advanced functionality types such as Theory of Mind (ToM) and self-aware AI represent ambitious goals for AI researchers. ToM AI would be capable of interpreting patient emotions, intentions, and psychological states, offering a new dimension of empathic and context-aware care [32]. This is particularly valuable in domains such as mental health, pediatrics, or palliative care, where human factors and emotional hints significantly influence treatment outcomes. Although prototypes exist in controlled environments, no fully operational ToM AI has been deployed clinically due to challenges in data representation, social cognition modeling, and trustworthiness [33].

Self-aware AI, which would possess consciousness and the ability to self regulate, remains purely speculative. While intriguing for future possibilities like autonomous surgical planning or independent disease research, it raises complex ethical and legal concerns about agency, liability, and control [34].

The convergence of AI capabilities and functionalities enables increasingly sophisticated healthcare systems. Federated learning is a form of collaborative ML without centralized data collection and has been employed to train AI models across multiple hospitals while preserving patient privacy [35,36]. Similarly, blockchain technology is being integrated with AI to ensure data transparency, traceability, and security in smart healthcare infrastructures [37].

In summary, AI is not a monolithic tool but a complex discipline with varying levels of intelligence and operational mechanisms. Understanding AI in healthcare through both capability-based and functionality-based lenses allows for a more evaluation of its current maturity, safety, and future trajectory. As we transition toward increasingly autonomous and intelligent systems, it becomes essential to balance innovation with ethical safeguards, ensuring AI continues to serve the core goal of healthcare improving human well-being.

### 1.1. Scope

This review focuses on the integration of AI into smart healthcare systems, specifically from two distinct yet complementary perspectives: AI capabilities and AI functionalities. The scope encompasses recent advances in AI technologies deployed for diagnosis, monitoring, treatment planning, mental health support, and personalized medicine. It covers real-world applications, ongoing research, and theoretical developments within the healthcare domain, emphasizing AI systems developed and implemented since 2021. The review spans across both clinical care (e.g., imaging, triage, and predictive modeling) and patient-centered applications. Table 1 shows the uniqueness of this review with other reviews.

### 1.2. Purpose

**Figure 2:**
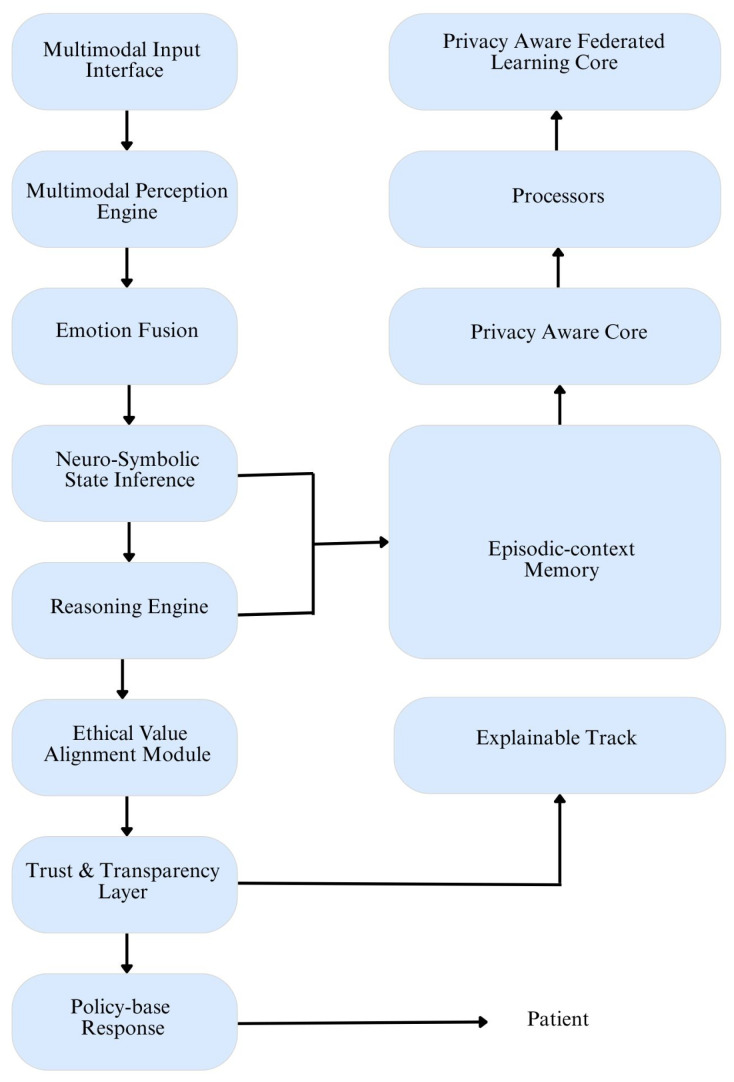
Proposed architecture of a General Artificial Intelligence (AGI) system for smart healthcare. The system integrates multimodal inputs (e.g., speech, vision, and physiological signals) through a perception engine, followed by emotion fusion and neuro-symbolic state inference. Episodic-context memory allows longitudinal tracking, while reasoning and ethical alignment modules ensure explainability, transparency, and value-sensitive behavior. Privacy-aware federated learning and explainable AI components enable secure, personalized, and human-centric policy-based responses for patient care.

The primary purpose of this review is to provide a structured and comprehensive understanding of how AI is being utilized in smart healthcare, categorized by its levels of intelligence (capabilities) and operational mechanisms (functionalities). Existing reviews often focus on specific technologies or applications without clearly distinguishing between different types of AI maturity or behavior. Figure 2 shows an AGI architecture for smart healthcare.

It integrates multimodal inputs, neuro-symbolic reasoning, and emotion fusion to support ethical, explainable, and trustworthy patient care. This paper fills that gap by the following:

- Clarifying the capability spectrum from narrow to Superintelligent AI.
- Outlining the functional evolution from reactive machines to theoretical self-aware systems.
- Mapping current technologies to these categories to evaluate readiness, risk, and research opportunities.

This framework helps healthcare professionals, AI developers, researchers, and policymakers better understand what AI can do today, what it may be capable of tomorrow, and how to design and regulate its use responsibly.

### 1.3. Contributions

This paper makes the following key contributions to the literature on AI in healthcare:

- Dual Perspective Framework: Introduces a unique classification of AI systems in smart healthcare based on capability (Narrow AI, General AI, and Superintelligence) and functionality (Reactive Machines, Limited Memory, ToM, and Self-Aware AI).
- Technology to Function Mapping: Provides a clear mapping of existing AI applications such as diagnostic imaging, predictive modeling, and AI mental health tools onto the defined capability and functionality axes.
- Contemporary Literature Synthesis (Post-2021): Consolidates and critiques recent research (2021–2025), including state-of-the-art techniques like federated learning, multimodal analysis, and AI power patient monitoring systems.
- Future Outlook and Ethical Insights: Highlights the ethical, legal, and operational challenges that arise as healthcare transitions toward more intelligent and autonomous AI systems, especially those approaching AGI or Superintelligent AI.
- Guidance for Stakeholders: Offers practical insights for healthcare practitioners, technologists, and policymakers to evaluate AI readiness, align it with clinical goals, and anticipate regulatory needs.

## 2. Methods

**Figure 3:**
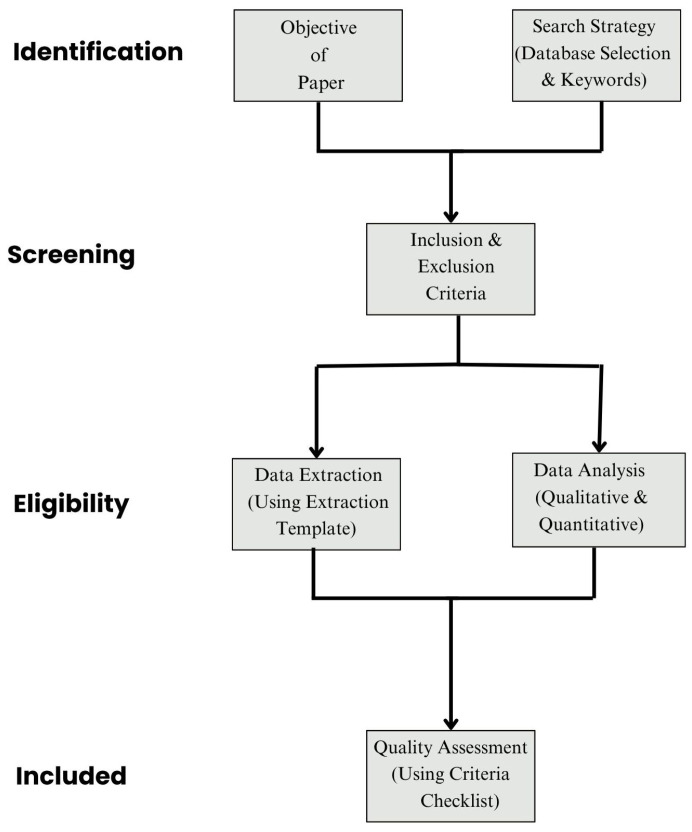
It highlights a structured approach from defining research objectives and search strategies to applying selection criteria, extracting and analyzing data, and conducting quality assessment.

This review was designed to provide a structured and comprehensive synthesis of the current state of Artificial Intelligence (AI) in smart healthcare systems, from the dual perspectives of AI capabilities and functionalities. Although not a systematic review, this study followed rigorous selection, categorization, and quality evaluation protocols inspired by the PRISMA guidelines in Figure 3 to ensure transparency and applicability.

### 2.1. Search Strategy

A comprehensive literature search was performed using four major academic databases: *PubMed*, *IEEE Xplore*, *Scopus*, and *Web of Science*. The search covered publications from January 2021 to May 2025 to capture the recent trends, developments, and deployments. Boolean keyword combinations were applied across titles, abstracts, and keywords:

- (“Artificial Intelligence” OR “AI” OR “machine learning” OR “deep learning” OR “generative AI”)
- AND (“smart healthcare” OR “clinical decision support” OR “digital health” OR “medical AI”)
- AND (“capabilities” OR “functionalities” OR “narrow AI” OR “AGI” OR “superintelligence” OR “Theory of Mind” OR “self-aware AI”)

Search results were exported into Rayyan for deduplication and initial screening. Citation chaining was also performed to include high-impact studies referenced in key articles.

### 2.2. Eligibility Criteria

Inclusion Criteria:

- Peer-reviewed journal or conference papers published in English between 2021 and 2025.
- Studies focused on AI applications in healthcare using clearly defined AI systems or frameworks.
- Articles discussing AI classification, capability levels (e.g., Narrow AI and AGI), or system functionalities (e.g., Limited Memory and ToM).
- Papers describing real-world or simulated deployment in clinical settings or smart healthcare infrastructure.

Exclusion Criteria:

- Non-peer-reviewed literature (e.g., preprints and whitepapers).
- Editorials, opinion pieces, or theoretical articles without application relevance.
- Studies outside the healthcare domain or focused solely on mathematical formulations of AI.
- Redundant studies not offering unique contribution to either capability-based or functionality-based classification.

### 2.3. Study Selection Process

The initial search yielded 800 unique records. After removing 278 duplicates, 522 studies were screened by title and abstract. Of these, 127 full-text articles were assessed for relevance to the dual classification framework, resulting in 84 studies being included in the final synthesis (Table 2). Discrepancies during selection were resolved by two independent reviewers and a third arbitrator.

### 2.4. Data Extraction and Mapping Framework

A structured extraction framework was developed to categorize studies based on AI capability and functionality dimensions. Data were recorded in a spreadsheet with the following variables (Table 3).

### 2.5. Quality Assessment Criteria

To ensure scientific rigor, we used an 8-point quality appraisal checklist adapted from existing frameworks such as TRIPOD-AI and DECIDE-AI:

1. Clear description of AI system and model architecture;
2. Defined clinical objective or healthcare application;
3. Description of data types and sources;
4. Explanation of capability or functionality alignment;
5. Evaluation of model performance or deployment outcome;
6. Evidence of clinical relevance or simulation;
7. Addressing of ethical or interpretability considerations;
8. Reproducibility elements (e.g., code availability and data links).

### 2.6. Data Synthesis Strategy

Instead, the data synthesis approach was structured around the following:

- Categorization by AI capability: Narrow, AGI, or Superintelligent.
- Categorization by functionality: Reactive, Limited Memory, ToM, or Self-Aware.
- Use case alignment (e.g., mental health, diagnostics, imaging, robotic systems).
- Mapping technologies to the dual framework.
- Thematic clustering of ethical and deployment challenges.

**Figure 4:**
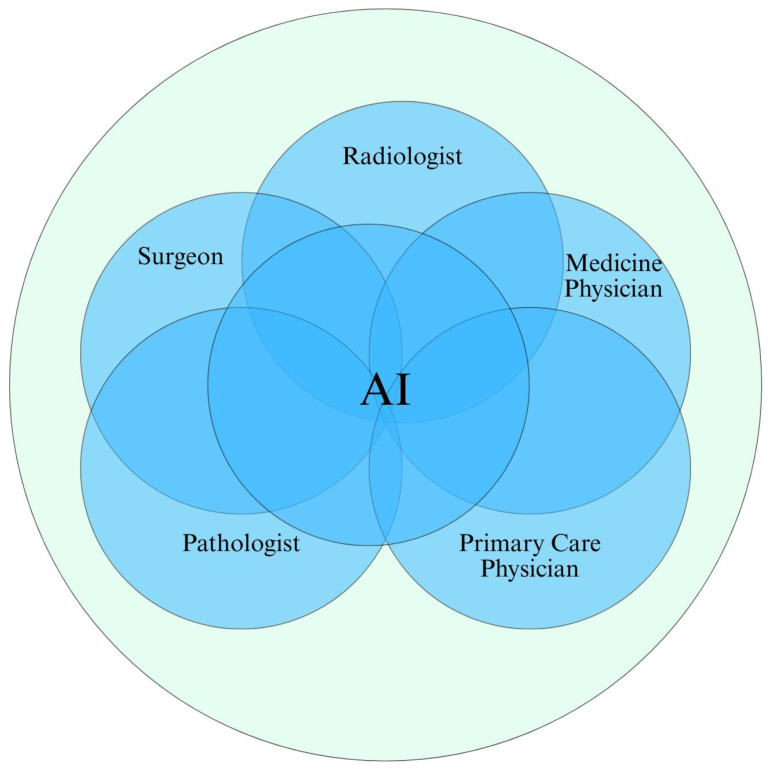
Collaborative integration of AI across clinical specialties. The Venn diagram illustrates how AI functions at the intersection of key medical roles—radiologist, pathologist, surgeon, medicine physician, and primary care physician. AI supports each domain through diagnostic assistance, image interpretation, treatment planning, and decision support, fostering a multidisciplinary approach to smart healthcare.

**Figure 5:**
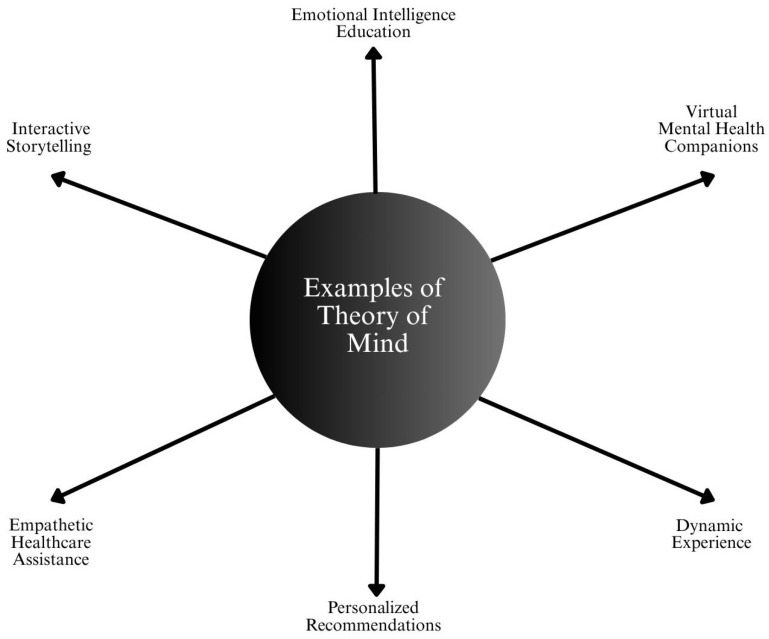
ToM applications in AI-driven healthcare and human–computer interaction. These include virtual mental health companions, empathetic healthcare assistance, personalized recommendations, emotional intelligence education, interactive storytelling, and dynamic patient engagement. ToM-enabled systems aim to understand and respond to user emotions, intentions, and social cues, enhancing contextual and human-centered care.

Descriptive statistics, comparative tables, and visual summaries (e.g., Figure 2, Figure 4, and Figure 5) were used to aid interpretation.

Software and Tools: Rayyan 1.6.1 (screening), Microsoft Excel version 2406 (extraction), Python 3.11.5 (analytics), LaTeX 2024 (reporting, visuals).

## 3. AI in Smart Healthcare: Based on Capabilities

AI has emerged as one of the most transformative forces in modern healthcare, enabling machines to analyze data, interpret medical information, assist clinicians, and even engage with patients. One meaningful way to classify AI systems is based on their capabilities, which refer to the breadth and depth of tasks the system can perform ranging from highly specialized, task-specific systems (Narrow AI), to generalized systems with human-like cognition (AGI), and ultimately, theoretical systems surpassing human intelligence (Superintelligent AI). Figure 4 shows multimodality and discipline in the healthcare system, connected with an AI system.

Understanding AI in healthcare through this capability-based framework provides a clear lens for evaluating technological readiness, clinical application, and system integration. Each level of capability carries distinct characteristics, scope of use, and performance paradigms that shape how AI is deployed in clinical practice. Table 4 shows the summary of AI in smart healthcare: based on capabilities.

### 3.1. Narrow AI: The Present Foundation of Smart Healthcare

Narrow AI, also known as Weak AI, refers to AI systems that are trained and optimized to perform a single, well-defined task. These systems do not possess consciousness, self-awareness, or general reasoning capabilities. Instead, they excel in pattern recognition and prediction within a confined problem space [40,41].

In healthcare, Narrow AI currently dominates AI applications. These systems power a wide array of diagnostic tools, decision support systems, chatbots, imaging software, and administrative automation tools [42,43].

Although tools like Wysa and Woebot have gained traction in English-speaking countries, deploying similar systems in Korea involves unique cultural and linguistic considerations. Korean language emotional recognition in NLP is still evolving, and public skepticism about AI-delivered psychological care persists. However, with growing mental health awareness and national support for digital therapeutics, localized chatbot platforms potentially trained on Korean clinical and linguistic are likely to emerge as scalable, stigma-reducing tools [44].

AI triage systems like Aidoc, although approved in several Western markets, face hurdles in Korea due to stricter medical device approval processes and non-standardized PACS (Picture Archiving and Communication System) integration across hospitals. For real-world adoption, these tools would require domestic validation trials, integration with EMR standards defined by Korean Health IT frameworks, and possible amendments to reimbursement codes that cover AI-based decision support tools [45].

#### 3.1.1. Applications in Medical Imaging and Diagnostic

One of the most successful applications of Narrow AI is in medical image analysis. Convolutional neural networks (CNNs) have demonstrated near human performance in tasks such as identifying pneumonia in chest X-rays, breast cancer in mammograms, and retinal abnormalities in fundus images [46,47,48,49]. These AI models are trained on labeled datasets and can process thousands of images in seconds, offering support for early diagnosis and reducing radiologist workload [50].

AI tools like Google’s DeepMind have developed models to detect over 50 eye diseases with accuracy comparable to expert ophthalmologists. Similarly, commercial solutions such as Aidoc and Zebra Medical Vision are integrated into hospital systems for triage and the detection of conditions like intracranial hemorrhage and pulmonary embolism [51].

#### 3.1.2. Clinical Decision Support Systems (CDSS)

Narrow AI systems also serve in clinical decision support, where they assist physicians by recommending treatments or identifying risk factors based on patient records. These systems typically employ ML algorithms trained on structured datasets like electronic health records (EHRs) [52]. For instance, models can predict the likelihood of sepsis, mortality, or hospital readmission, aiding clinicians in proactive care planning [53].

#### 3.1.3. Virtual Health Assistants and Chatbots

AI-powered chatbots such as Woebot, Wysa, and Tess are being widely used in mental health contexts. These tools use natural language processing (NLP) and predefined conversation flows to deliver cognitive behavioral therapy (CBT), monitor mood, and offer coping strategies. Ref [34] reported that such chatbots have shown promising results in reducing symptoms of depression and anxiety while maintaining high user engagement [54].

#### 3.1.4. Wearable Devices and Remote Monitoring

Narrow AI is also embedded in wearable healthcare devices, including smartwatches and fitness bands. These devices monitor heart rate, oxygen saturation, sleep cycles, and more. AI algorithms analyze these signals to detect arrhythmias, track stress levels, or predict potential complications in chronic disease patients. This capability is increasingly used for remote patient monitoring, enabling early interventions and reducing hospital admissions [55,56].

#### 3.1.5. Administrative and Workflow Optimization

In addition to clinical tasks, Narrow AI systems support hospital operations. Natural language processing models are used to summarize clinical notes, manage medical billing, and automate appointment scheduling, contributing to operational efficiency.

While the performance of Narrow AI is typically limited to the domain for which it was trained, its widespread adoption underscores its reliability and practicality in routine medical functions. It represents the foundation of today’s smart healthcare infrastructure [57,58].

### 3.2. General AI: Toward Contextual and Adaptive Intelligence

General AI, also known as Strong AI or AGI, refers to AI systems with human-like cognitive capabilities, and the ability to understand, learn, and apply knowledge across a wide range of tasks. Unlike Narrow AI, AGI is not constrained by task specificity; it can adapt to new situations, understand context, and engage in abstract reasoning.

In healthcare, the concept of AGI evokes the image of an AI-powered clinician who can synthesize clinical notes, lab results, genetic profiles, and real-time patient data, and then recommend or even explain optimal treatment strategies with empathy and contextual awareness [12,59].

#### 3.2.1. Multimodal Patient Understanding

Efforts to develop AGI are rooted in the goal of integrating heterogeneous data sources including imaging, lab values, genomics, lifestyle information, and historical health data into unified patient representations. This would allow AI to approach diagnosis and treatment planning from a holistic perspective, akin to a skilled physician.

Some of the early movement toward this capability can be seen in large language models (LLMs) and foundation models. Tools like ChatGPT (GPT-4), Med-PaLM 2, and GatorTron-large have demonstrated the ability to understand clinical language, generate medical advice, and even answer board-level medical exam questions with high accuracy [60].

These LLMs, when fine-tuned with clinical data, can synthesize long documents, answer contextual queries, and adapt explanations to different audiences (e.g., patients vs. doctors). Although they do not yet qualify as AGI, they represent a major step forward in general purpose medical reasoning [61].

#### 3.2.2. Cognitive Flexibility in Mental Health Applications

Another emerging application of AGI principles is in personalized mental health care. For example, AI systems designed to understand emotional context and deliver real-time support are being enhanced to adjust their behavior based on user personality, history, and engagement trends [62].

Lee et al., [53] highlighted that as LLMs gain conversational depth, they may serve as empathy-aware agents, dynamically tailoring their responses to patient emotional states. This type of context-aware interaction is a defining trait of AGI.

#### 3.2.3. Adaptive Learning in Clinical Settings

Some AI research systems are exploring adaptive learning, where models continuously update themselves based on new data without retraining from scratch. This could enable the development of AI tools that stay current with evolving clinical guidelines and patient populations, a core feature of AGI in practice [63].

**Figure 6:**
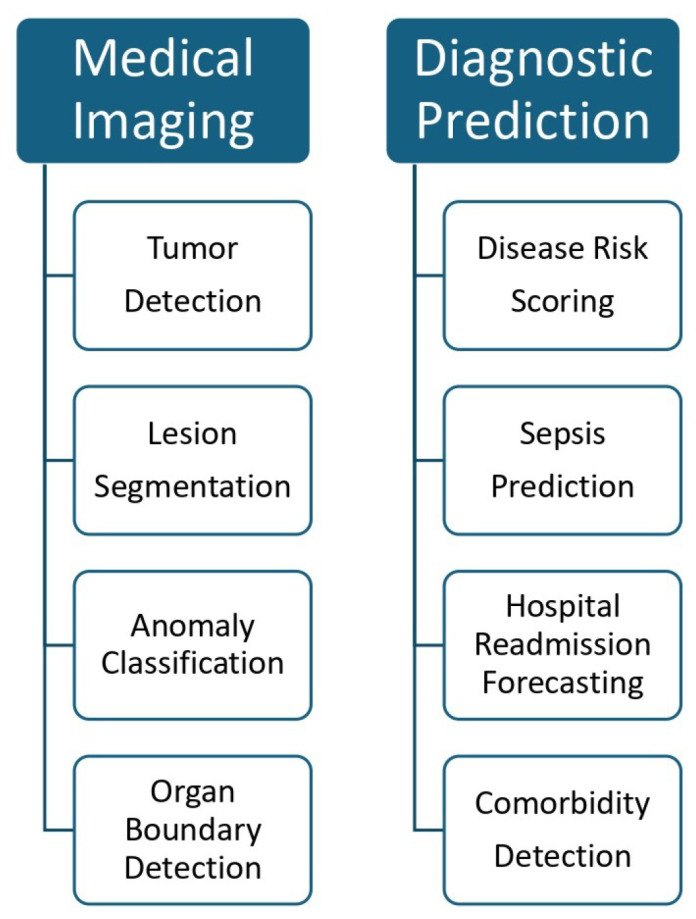
Representative applications of AI in medical imaging and diagnostic prediction. On the left, AI-driven medical imaging tasks include tumor detection, lesion segmentation, anomaly classification, and organ boundary detection. On the right, diagnostic prediction applications include disease risk scoring, sepsis prediction, hospital readmission forecasting, and comorbidity detection. These use cases demonstrate how AI enhances precision and efficiency in both image-based and data-driven clinical workflows.

While no fully functioning AGI system is operational in healthcare today, existing systems are progressively demonstrating task generalization, multimodal integration, and contextual adaptability, characteristics associated with this capability level [64]. Figure 6 shows an AI application in medical imaging and its diagnosis.

### 3.3. Superintelligent AI: Theoretical Cognitive Supremacy

Superintelligent AI refers to hypothetical AI systems whose intellectual capacities far exceed those of the most capable human minds in every domain, including medicine, ethics, scientific discovery, and emotional intelligence [65].

In the context of smart healthcare, Superintelligent AI would possess the ability to outperform top-tier medical specialists, predict disease outbreaks before they occur, design new drugs or therapies autonomously, and manage global healthcare systems with unmatched precision and efficiency [66].

#### 3.3.1. Autonomous Knowledge Discovery

A Superintelligent AI system in medicine could autonomously perform the following tasks:

- Read and synthesize thousands of new research papers daily.
- Design novel clinical trials.
- Model the effects of drugs at the molecular level.
- Devise treatment plans personalized to the genetic and epigenetic profile of each individual.

Such systems would likely be equipped with advanced versions of today’s AI technologies, enhanced by recursive self-improvement mechanisms and long-term goal orientation.

#### 3.3.2. Global Health System Management

Beyond individual patient care, Superintelligent AI could potentially manage healthcare systems at scale. It could optimize resource distribution across countries, forecast and contain pandemics, and even make high-stakes decisions in complex bioethical scenarios with precision and fairness [67].

Bostrom et al., [68] argues that such a system could also contribute to meta-research, designing and executing new methodologies in clinical science or even identifying biases in current medical knowledge frameworks.

#### 3.3.3. Integration of Ethical, Emotional, and Social Intelligence

Superintelligent AI may exceed human capability not only in analysis but also in emotional intelligence, managing doctor–patient conversations more compassionately, understanding diverse cultural contexts, and engaging in therapeutic dialogues more effectively than any human practitioner [69].

Morley et al., [70] notes that discussions around AI in healthcare must include this potential, particularly for informing regulatory frameworks and responsible innovation principles.

While this capability level remains hypothetical, it is often used in academic and policy circles as a benchmark to guide AI alignment, accountability structures, and technological boundaries in healthcare [71].

## 4. AI in Smart Healthcare: Based on Functionalities

In the evolving landscape of smart healthcare, AI is not only categorized by its level of intelligence (capabilities) but also by how it functions—its architecture, operational logic, and decision-making paradigm. A classification based on AI functionalities provides essential insights into how AI systems operate within the clinical settings. This approach is rooted in cognitive science and engineering, and it helps distinguish AI systems by their ability to interact with and learn from the environment [72,73].

This classification divides AI into four primary functional types:

1. Reactive Machines.
2. Limited Memory.
3. Theory of Mind.
4. Self-Aware Systems.

Each category represents a higher level of complexity in terms of perception, processing, and interaction. Functional classification is especially relevant to healthcare, where AI must often perform high-stakes decisions based on partial, multimodal, or temporal data, while also engaging with clinicians and patients.

Table 5 shows a summary of the functionality-based classification of AI in smart healthcare.

### 4.1. Reactive Machines

Reactive machines represent the most elementary type of AI. These systems do not possess memory or an internal understanding of the world. They operate purely on real-time input and are unable to learn from historical data [74]. Their responses are deterministic and based on predefined rules or heuristics.

#### 4.1.1. Structure and Operation

Reactive AI systems are often rule-based and implement decision trees or logical sequences to produce outputs in response to specific inputs. They are typically embedded in hardware systems or limited-function software applications [75].

#### 4.1.2. Applications in Smart Healthcare

Although limited in flexibility, reactive machines have found meaningful application in various components of modern healthcare:

- ICU Alarm Systems: These systems detect abnormal parameters in patient vitals, such as heart rate or oxygen saturation, and trigger alerts. They follow pre-set thresholds and act instantaneously without learning from past cases [76].
- Early Expert Systems: Tools like MYCIN (for infectious diseases) and Internist-I (for internal medicine) are classic examples of reactive systems in medicine. These systems used if–then logic to provide diagnostic suggestions and therapeutic options [77].
- Medical Device Automation: Many medical machines like infusion pumps, ventilators, and defibrillators rely on reactive logic to function safely in real time without adapting from previous data [78].

#### 4.1.3. Value in Healthcare

Despite their lack of adaptability, reactive machines offer high reliability, speed, and interpretability. They are especially useful in environments where consistency and real-time responsiveness are more important than adaptive intelligence [79].

### 4.2. Limited Memory Systems

The majority of modern AI applications in healthcare fall under the category of limited memory systems. These systems are capable of accessing past information, either directly (e.g., patient history) or indirectly (e.g., training data), to make decisions. However, they do not learn continually or autonomously from new experiences beyond retraining cycles [80].

#### 4.2.1. Architecture

Limited memory systems typically involve supervised or semi-supervised ML models trained on large datasets. They include both classical ML algorithms and DL architectures [81].

#### 4.2.2. Applications in Smart Healthcare

This category encompasses a broad range of impactful applications:

- Medical Imaging: AI models using deep CNN are widely used for detecting tumors, lesions, and organ anomalies from radiographic images. Ref. [82] showed how hybrid CNN-based systems accurately predicted breast cancer metastasis from mammograms and metadata.
- Risk Stratification: ML models trained on electronic health records (EHRs) can predict hospital readmission, mortality, or sepsis development. These models consider past diagnoses, medications, and lab results to generate risk scores [53].
- Wearable Monitoring and Remote Sensing: Devices like Fitbit, Apple Watch, or specialized ECG patches use AI to monitor physiological signals such as heart rate, sleep cycles, or respiratory rate. These tools analyze patterns over time and alert users or providers about concerning trends [83].
- Digital Mental Health Tools: Chatbots such as Woebot and Wysa employ session-based memory to deliver tailored psychological interventions. They remember user inputs during a session to provide context-aware dialogue and offer real-time cognitive behavioral therapy [34].
- Drug Discovery and Genomics: AI is used to analyze genomic sequences and predict drug target interactions based on historical molecular data [22,84]. These models improve over time as more training data is incorporated during periodic updates [85].

#### 4.2.3. Functional Characteristics

- Utilizes stored data for prediction.
- Requires retraining for model updates.
- Supports short-term memory within fixed boundaries.
- Does not generalize across tasks.

Limited memory systems form the core operational tier of today’s smart healthcare applications. They are trusted, data-based tools embedded across imaging, diagnostics, monitoring, and virtual care [86,87].

### 4.3. Theory of Mind Systems

ToM in AI refers to the ability of systems to model human emotions, intentions, beliefs, and social cues. This level of functionality is inspired by cognitive psychology, where ToM is central to empathetic and cooperative behavior [88].

Although no fully operational ToM AI exists, early prototypes and research systems demonstrate partial capabilities, particularly in mental health, elder care, and human–AI interaction design [89]. Figure 5 shows an example of ToM with AI in healthcare.

#### 4.3.1. Operational Features

A Theory of Mind AI performs the following tasks [90,91]:

- Infers user intent beyond text or data.
- Understands affective states and behavioral context.
- Adjusts responses based on the perceived emotional or cognitive status of the user.

#### 4.3.2. Healthcare Applications

- Empathy-Aware Mental Health Tools: AI chatbots enhanced with emotion recognition capabilities can detect user tone, sentiment, or emotional distress. The EmpatheticDialogues dataset and systems trained on it are being explored for empathetic response generation [92].
- Conversational AI in Counseling: Advanced NLP systems are being adapted for therapy bots that can adjust interaction styles based on patient emotional feedback. Ref. [34] reported that users prefer bots that demonstrate empathy, mirroring basic Theory of Mind behavior.
- Pediatric and Geriatric Care Assistants: In environments where patients may be non-verbal or cognitively impaired, AI systems using facial expression and speech pattern recognition can infer emotional or physical discomfort [92].
- Clinical Communication Support: Systems are being designed to assist doctors in delivering complex or sensitive information, with AI suggesting language modifications based on the patient’s comprehension level and psychological state [93].

#### 4.3.3. Multimodal Fusion for ToM

Achieving ToM requires AI to combine multiple data types:

- Text (conversation).
- Audio (tone, pitch, emotion).
- Visual (facial expression, body language).
- Contextual data (history, environment).

Lee et al., [53] emphasized that such systems are critical for context-aware human and computer interaction, particularly in mental health, where personalization and empathy are central to treatment adherence.

Although not yet mainstream in clinical deployment, these systems are at the forefront of human-centered AI in healthcare.

### 4.4. Self-Aware Systems

Self-aware AI represents the highest and most complex form of functional intelligence. These systems would possess not only ToM but also consciousness, and the ability to model their own state and adapt behavior in real time, based on introspection.

While no self-aware AI exists, certain design features are being explored in advanced AI research that mimic aspects of self-awareness relevant to healthcare safety and performance [94].

#### 4.4.1. Emerging Concepts in Healthcare Systems

- Explainable AI (XAI): Systems that provide rationales for their decisions, particularly in medical imaging or diagnosis. Saliency maps in CNNs highlight which part of an X-ray image influenced the model’s decision early form of self-reflective behavior [60].
- Uncertainty Estimation: AI models that can indicate when they are not confident in a prediction simulate a rudimentary form of introspection [95].
- Adaptive Clinical Learning Systems: Systems that monitor their own performance across populations, and suggest re-training or flag anomalous data points, embody limited aspects of meta cognition [96].

#### 4.4.2. Application in Risk Management

Self-aware functionality is being integrated into clinical AI monitoring systems. These systems log all predictions, flag inconsistencies, and alert human supervisors to anomalies, forming a loop of machine accountability. This is particularly relevant in radiology, intensive care, and automated drug dosing platforms [39].

#### 4.4.3. Therapeutic Identity in Mental Health AI

Some developers are exploring whether long-term therapeutic AI companions should maintain a consistent emotional identity, remember past conversations, and express continuity in care. Although these systems are not conscious, they simulate memory and personality to enhance therapeutic rapport.

While fully self-aware AI remains hypothetical, elements of meta learning, decision traceability, and confidence estimation are becoming integral to building trustworthy and safe AI in healthcare [97].

## 5. Synthesis: Capabilities vs. Functionalities

To comprehensively understand the role and trajectory of AI in smart healthcare, it is essential to synthesize the two primary frameworks used to evaluate AI: capability-based classification and functionality-based classification. While capability-based analysis focuses on the level of intelligence (Narrow AI, AGI, and Superintelligent AI), functionality-based analysis addresses how the AI operates (Reactive Machines, Limited Memory, ToM, and Self-Aware Systems). By aligning these two frameworks, we can more accurately evaluate the current state of AI systems, identify their clinical utility, and conceptually map the intersections of intelligence level and operational design. Table 6 shows the integrative perspectives of AI capabilities and functionalities in smart healthcare.

This section provides a structured synthesis of these dimensions by examining three strategic intersections:

1. Narrow AI + Limited Memory.
2. AGI + Theory of Mind.
3. Superintelligent AI + Self-Awareness.

### 5.1. Narrow AI + Limited Memory: The Operational Backbone of Today’s Smart Healthcare

The intersection of Narrow AI (task-specific intelligence) and Limited Memory (use of historical data without continuous learning) forms the foundation of current AI implementations in smart healthcare. These systems dominate the clinical landscape because they strike a practical balance between performance, interpretability, and feasibility.

#### 5.1.1. Current Use in Smart Healthcare

Narrow AI with Limited Memory functionality is utilized across numerous domains:

- Clinical Decision Support Systems (CDSS): Tools that provide physicians with evidence-based suggestions for diagnosis and treatment based on structured data from EHRs [98].
- Medical Imaging: CNN-based models trained to detect abnormalities such as tumors, fractures, or nodules from CT, MRI, and X-ray images [99].
- Predictive Analytics: Algorithms that forecast risks of readmission, sepsis, or treatment complications using past patient data [100].
- Mental Health Chatbots: Tools like Wysa and Woebot use session-based memory and NLP to offer CBT and mood tracking [101].

These systems work with large but finite datasets, operate within strict task boundaries, and improve performance through retraining rather than through real-time experiential learning.

#### 5.1.2. Value Proposition

- High accuracy within specialized domains.
- Trustworthy through auditability and static behavior.
- Relatively low risk in deployment due to limited autonomy.

This pairing represents a mature, clinically validated category of AI tools that are routinely deployed in hospitals, telehealth platforms, diagnostic centers, and mental health applications [39].

### 5.2. General AI + Theory of Mind: The Emerging Horizon of Adaptive, Empathetic Intelligence

The conceptual pairing of AGI (systems with human-level generalization and reasoning) with ToM (understanding of human beliefs, emotions, and intentions) reflects the next frontier of AI in healthcare. Though not yet realized, this intersection represents the vision for context-aware, multimodal, and emotionally responsive AI systems.

#### 5.2.1. Current Use in Smart Healthcare

Currently, there are no fully operational systems at the AGI + ToM level. However, precursors exist:

- Large Language Models (LLMs): Systems like Med-PaLM and GatorTron exhibit early-stage general reasoning capabilities across diverse clinical queries [102].
- Multimodal AI Models: Research is underway to integrate imaging, EHR data, genomic profiles, and behavioral metrics into unified decision-making tools [103].
- Affective Computing: Emotion-aware chatbots and assistive robots that respond to user tone and sentiment are early steps toward ToM in AI [104].
- Contextual Care Tools: Systems designed to adapt communication style depending on whether the user is a clinician, caregiver, or patient [53].

#### 5.2.2. Functionality and Potential

These systems are envisioned to perform the following tasks:

- Handle unstructured and multimodal data.
- Understand the mental state and intent of the use.
- Adjust behavior based on empathy, cultural awareness, and situational context.

This intersection marks the transitional zone from utility-focused AI to patient-centered AI, where the system’s intelligence is not just in its accuracy but also in its ability to engage and collaborate [105,106].

#### 5.2.3. Representative Clinical Use Cases for AGI in Smart Healthcare

While AGI remains largely conceptual, its envisioned capabilities such as multimodal reasoning, contextual learning, and adaptive decision-making can be better appreciated when situated within specific healthcare scenarios. Below, we outline three representative clinical use cases where AGI could deliver transformative impact by synthesizing diverse data sources and making context-aware decisions in real time [29,107].

In critical care settings, patient conditions can change rapidly, requiring the continuous integration of high-frequency data such as vital signs, lab results, imaging, physician notes, and ventilator parameters. An AGI-enabled system could act as an intelligent clinical assistant, recognizing subtle trends that precede deterioration (e.g., sepsis and cardiac arrest) and proposing early interventions. It could prioritize tasks, alert clinicians, and simulate the possible outcomes of different treatment paths, enhancing safety and precision in time-sensitive environments.

In rural clinics or underserved regions with limited access to specialists, AGI systems could assist in diagnosing patients based on limited but diverse data inputs such as patient history, verbal symptoms, basic imaging (e.g., portable ultrasound), and vital signs. By adapting to local languages, cultural contexts, and healthcare protocols, AGI could provide actionable insights for frontline healthcare workers. Such systems would support equitable access to care, while simultaneously learning from geographically diverse data distributions [53,108].

In biomedical research, AGI could analyze vast quantities of omics data (e.g., genomics and transcriptomics), clinical trial databases, and the latest literature to identify novel drug targets or repurpose existing therapeutics. In personalized medicine, AGI could generate patient-specific treatment plans by integrating molecular profiles with lifestyle data, imaging, and physician feedback. This scenario demonstrates the potential of AGI as both a scientific discovery engine and a clinical collaborator in precision oncology [30].

### 5.3. Superintelligent AI + Self-Awareness: A Theoretical Apex of Cognitive and Ethical Complexity

The highest conceptual intersection in the synthesis framework is that of Superintelligent AI (systems exceeding human intelligence in all areas) and self-awareness (systems that possess a sense of self and introspective reasoning). This represents a purely theoretical construct at present but is often used as a philosophical and strategic benchmark for understanding the limit and risks of AI in healthcare.

#### 5.3.1. Current Use in Smart Healthcare

There are no existing systems that embody both superintelligence and self-awareness in healthcare or any other domain. However, elements of this vision are explored through the following:

- Explainable AI (XAI): Systems that rationalize their own decisions (e.g., saliency maps and attention mechanisms) [109].
- Uncertainty Quantification: AI models that indicate the degree of confidence in their predictions, enabling human oversight [95].
- Self-Monitoring Agents: Systems capable of logging their performance, flagging anomalies, and recommending updates [110].

These features are components of a broader theoretical aspiration to build autonomous AI systems that can reflect, reason, and refine themselves continuously.

#### 5.3.2. Conceptual Role in Smart Healthcare

In theory, Superintelligent + Self-Aware AI would perform the following tasks:

- Independently conduct medical research and discover treatments.
- Run entire healthcare ecosystems autonomously.
- Resolve ethical dilemmas by weighing societal impact, cultural norms, and individual patient values.
- Provide lifelong, personalized care surpassing human limitations in cognition and availability.

Such a system would no longer serve merely as an assistant or collaborator but would emerge as an autonomous healthcare agent, capable of managing and optimizing care for entire populations while also customizing treatment at the individual level [111,112].

### 5.4. Comparative Framework: Bridging Capabilities and Functionalities

To visualize the intersection of these dimensions, the following table summarizes the synthesis:

#### Key Insights from the Synthesis

1. Most Deployed Systems Reside in the Narrow AI + Limited Memory Quadrant: These systems dominate because they are practical, validated, and easier to regulate, making them ideal for tasks like diagnostics and workflow automation [105].
2. Emerging Research Aligns with the AGI + Theory of Mind Paradigm: There is growing momentum toward creating emotionally intelligent and context-aware systems. While these models show promise, they require significant advancement in natural language understanding, multimodal processing, and interoperability [113].
3. Superintelligent + Self-Aware Systems Serve as a Theoretical Boundary: This quadrant is valuable for philosophical, ethical, and governance considerations, guiding the development of safeguards and frameworks even before such systems exist [114].

## 6. Challenges and Considerations in AI-Driven Smart Healthcare

While AI continues to revolutionize smart healthcare by enhancing diagnostic precision, streamlining clinical workflows, and enabling personalized treatment, the integration of AI technologies introduces several complex challenges and ethical considerations. These concerns span across technical, social, regulatory, and clinical domains, and they become more pronounced as AI systems evolve from task-specific tools to autonomous agents with greater decision-making influence.

This section outlines four key issues that must be addressed for AI to be deployed safely, ethically, and equitably in healthcare settings: bias and fairness, interpretability, regulation, and data security.

### 6.1. Bias and Fairness

One of the most pressing challenges in AI development is bias in data and models, which can lead to unfair or harmful outcomes, particularly for underrepresented groups.

#### 6.1.1. Sources of Bias

Bias can enter an AI system at multiple stages:

- Training Data Bias: When training datasets are skewed toward specific populations (e.g., white, male, and urban patients), AI models may underperform for marginalized communities. Dermatology AI trained on light-skinned images may fail to detect skin cancer in patients with darker skin tones [115].
- Labeling Bias: If clinical labels are assigned inconsistently by different practitioners, especially in subjective diagnoses (e.g., mental health and pain levels), AI systems may learn incorrect or misleading associations [116].
- Deployment Bias: Once deployed, AI tools may exacerbate disparities if they are more accessible to high-income or tech-savvy populations, leaving others underserved [117].

#### 6.1.2. Impact on Healthcare Equity

Bias in AI can have serious implications in healthcare [118,119], including the following:

- Misdiagnosis or missed diagnosis in minority populations.
- Allocation of resources skewed toward majority groups.
- Worsening of health disparities despite the promise pf AI to reduce them.

### 6.2. Interpretability and Trust

In healthcare, where decisions are often life-critical, clinicians must be able to understand and trust AI outputs. This raises the issue of interpretability, or the extent to which a human can comprehend how an AI system arrived at its conclusion.

#### 6.2.1. The “Black Box” Problem

Many high-performing AI models—particularly DL systems—operate as “black boxes”, where the internal decision-making process is opaque to users [120,121]. This lack of transparency can hinder clinical trust and acceptance, especially in the following cases:

- AI recommendations contradict clinical judgment.
- There are legal or ethical consequences for incorrect predictions.
- The user cannot justify an AI-driven diagnosis or treatment to the patient.

#### 6.2.2. Clinical Implications

Lack of interpretability can lead to the following outcomes [122,123] (Table 7):

- Delayed adoption of effective tools.
- Overreliance on AI without appropriate oversight.
- Resistance from clinicians due to lack of confidence.

### 6.3. Regulatory Complexity and Oversight

AI in healthcare operates at the intersection of technology, medicine, and law, and must therefore be governed by robust regulatory frameworks. However, current regulatory mechanisms were not designed for adaptive, data-based systems that can learn and evolve.

#### 6.3.1. Capability-Specific Regulation

AI systems at different capability levels demand different regulatory strategies:

- Narrow AI systems (e.g., imaging classifiers) can be regulated similarly to traditional medical devices through validation, accuracy thresholds, and risk assessments [129].
- AGI models (e.g., foundation models for diagnosis) require broader guidelines, especially for ethical alignment, training data provenance, and cross-context generalizability [130].
- Autonomous AI systems, as envisioned in superintelligence or advanced self-awareness, challenge current regulatory paradigms entirely and call for international coordination and ethical governance [131].

#### 6.3.2. Current Regulatory Bodies and Guidelines

- The FDA (U.S.) has begun to regulate AI/ML based Software as a Medical Device (SaMD), requiring manufacturers to provide evidence of performance, safety, and effectiveness [132].
- The European Union’s AI Act classifies healthcare AI as “high-risk”, mandating transparency, human oversight, and post-market monitoring [133].
- Global efforts, such as the WHO’s guidance on AI ethics in healthcare, are emerging to set universal standards [134].

### 6.4. Data Security and Privacy

The use of AI in healthcare requires access to vast amounts of sensitive patient data, including medical histories, genetic information, and real-time sensor data. This raises critical concerns around data privacy, security, and consent.

#### 6.4.1. Risks Involved

- Data breaches can lead to the exposure of personal health information (PHI), with legal and ethical consequences [135].
- Re-identification attacks may occur when anonymized datasets are matched with external data sources [136].
- Unauthorized model inference could allow third parties to extract sensitive information from AI systems, especially generative models [137].

#### 6.4.2. Increasing Risk with Advancing AI

As AI systems grow more capable, the risk of privacy violation increases [138,139]:

- Advanced models may memorize training data, especially if not properly regularized.
- Cloud-based AI platforms introduce vulnerabilities in data storage and access.
- Cross-institutional models, such as federated learning, while designed for privacy, still pose metadata leakage risks.

#### 6.4.3. Regional Feasibility of AGI Development: The Case of Korea

While this review discusses the potential of AGI in healthcare from a global perspective, it is important to contextualize its feasibility within the regional legal and data governance frameworks particularly in South Korea, where the authors are based. Developing and deploying AGI systems for smart healthcare requires large volumes of diverse, multimodal data, including medical imaging, clinical notes, genomic data, and real-time patient monitoring streams. However, access to such data is highly regulated by national laws.

In Korea, the Personal Information Protection Act (PIPA) is one of the strictest data privacy laws in Asia and imposes significant restrictions on the secondary use of personal and health-related data, even after de-identification [44]. Additionally, the Medical Service Act limits the sharing of medical records outside authorized institutions and requires strict consent procedures for research use [140]. These laws are designed to protect patient rights but can inadvertently hinder the large-scale data aggregation needed for training AGI and multimodal AI systems.

Moreover, Korea does not yet have comprehensive legal frameworks supporting data altruism, data trusts, or dynamic consent models, which are being piloted in the European Union under the GDPR umbrella [141,142]. Although there are emerging initiatives such as the “MyData Korea Project”, which aim to give individuals more control over their health and financial data, the ecosystem remains fragmented and lacks integration with AI research infrastructure [143].

From a technical standpoint, methods like federated learning and synthetic data generation have been proposed to work around data centralization issues [35]. However, these too must align with local encryption, anonymization, and data retention regulations. In Korea, federated learning in healthcare remains largely in pilot stages and faces interoperability challenges among hospitals with heterogeneous IT systems [27].

Therefore, while Korea holds substantial technological capabilities, its legal landscape currently limits AGI feasibility unless supported by regulatory innovation and international policy harmonization. We propose that future national strategies focus on the following:

- Establishing AI-specific ethical data governance frameworks.
- Encouraging privacy-preserving data sharing across medical institutions.
- Aligning domestic laws with global AI policy efforts to enable international collaboration.

### 6.5. Cross-Disciplinary Insights into Explainability, Fairness, and Robustness

While the challenges of bias, explainability, and reliability are critical within the domain of medical AI, they are not unique to healthcare. Other high-stakes fields such as finance, autonomous driving, and legal informatics have similarly grappled with the need for transparency, fairness, and robustness in AI-driven decision-making. Leveraging the lessons learned from these domains can offer meaningful strategies for healthcare-specific implementations.

In financial services, particularly in fraud detection and credit scoring, AI models are now required to provide interpretable outputs to comply with regulatory requirements such as the European Union’s GDPR and the U.S. Fair Credit Reporting Act. These systems often employ local surrogate models like LIME or SHAP, along with counterfactual explanations to make model predictions understandable to non-technical stakeholders [144,145]. A similar strategy could be applied in clinical diagnostics, where providing clinicians with localized visual or textual explanations (e.g., “feature X contributed Y% to the decision”) could enhance the trust and adoption of AI tools.

In legal technology and automated hiring systems, bias mitigation is critical due to the risk of algorithmic discrimination. These fields have developed techniques such as fairness-aware learning, adversarial debiasing, and preprocessing methods like re-weighting or re-sampling [146]. Translating these approaches into healthcare could help address disparities in diagnostic accuracy across demographic subgroups especially in areas like dermatology or radiology, where bias based on skin tone or socioeconomic status has been documented.

Autonomous driving systems must operate under unpredictable conditions and are therefore designed to detect “corner cases”: rare, high-risk scenarios that fall outside the training distribution. Strategies such as uncertainty quantification, anomaly detection, and redundancy layering have been widely implemented [147,148]. In healthcare, similar techniques could be adopted to flag atypical patient presentations (e.g., rare disease phenotypes or poly-morbid conditions) and trigger escalation to human review, enhancing safety in edge cases.

By adopting these cross-domain methodologies, medical AI systems can benefit from matured practices already tested in regulatory, real-time, or high-liability contexts. These strategies can enrich healthcare-specific frameworks in the following ways:

- Applying financial XAI techniques to enhance clinical interpretability and shared decision-making.
- Adapting legal fairness audits for demographic bias tracking in clinical trials and AI validation datasets.
- Utilizing robustness tools from autonomous systems to manage uncertainty and atypical patient cases.

This interdisciplinary convergence fosters a more holistic and responsible approach to developing medical AI systems that are not only technically accurate, but also ethically aligned, transparent, and resilient in real-world environments.

### 6.6. Limitations

Despite the comprehensive approach adopted in this review, several limitations must be acknowledged to ensure an accurate and balanced interpretation of the findings.

Language and Publication Bias: This review included only peer-reviewed articles published in English between January 2018 and May 2025. As a result, important studies reported in non-English journals or local repositories may have been excluded, particularly those from countries with strong domestic AI development (e.g., China, Japan, and Korea) but limited English-language dissemination. This may have introduced a language bias and reduced the global representativeness of the findings.

Database and Search Scope: Although the search strategy covered four major databases (PubMed, IEEE Xplore, Scopus, and Web of Science), there remains the possibility that relevant research indexed in discipline-specific or regional databases (e.g., CNKI or KoreaMed) was overlooked. Moreover, gray literature, industry white papers, and technical documentation from commercial AI vendors were not included, which may limit insights into real world deployment beyond academic settings.

Heterogeneity of Included Studies: The studies included in this review varied widely in terms of the clinical domain, data modality, AI model used, and evaluation metrics. Due to this heterogeneity, it was not possible to conduct a quantitative meta-analysis. Instead, we relied on narrative synthesis and descriptive statistics, which may be more subjective and less robust than statistical aggregation.

Limited Generalizability Across Regions: While internationally recognized AI solutions such as DeepMind, Aidoc, and Wysa were highlighted, their feasibility and impact were not uniformly evaluated across all healthcare systems. For example, only a limited contextual comparison was made with Korea’s healthcare infrastructure, legal restrictions, and digital readiness. This may reduce the policy relevance for countries with differing levels of AI maturity, regulatory constraints, or cultural attitudes toward medical technology.

Rapid Technological Advancements: The field of Artificial Intelligence, particularly generative models and AGI-related architectures, is evolving at an accelerated pace. Some of the technologies discussed may already be superseded or improved upon by newer frameworks by the time of publication. Consequently, while the review reflects the state of the art as of mid 2025, it may not fully capture the most recent developments in this dynamic field.

Underreporting of Clinical Deployment Outcomes: Many of the reviewed studies focused on algorithm development, retrospective validation, or simulation-based performance. Relatively few reported on real-world clinical deployment, long-term patient outcomes, or user acceptance by healthcare providers. This limits our ability to draw conclusions about the safety, scalability, and ethical impact of AI systems in live healthcare environments.

Incomplete Evaluation of Explainability and Fairness: Although fairness and interpretability were key inclusion criteria, many studies lacked standardized methods to assess these properties. Some relied solely on post hoc techniques (e.g., SHAP and Grad-CAM) without evaluating clinician comprehension, trust, or diagnostic confidence. Additionally, few studies disaggregated performance by patient subgroups (e.g., race, gender, and socioeconomic status), raising concerns about unaddressed algorithmic bias.

AGI-Specific Limitations: The discussion on AGI was primarily conceptual, due to the lack of real-world AGI implementations in healthcare. While use cases were hypothesized based on trends in multimodal learning and generalist models (e.g., Med-PaLM), no empirical studies currently demonstrate AGI capabilities in clinical settings. Thus, projections made regarding the AGI integration remain speculative and must be interpreted with caution. Lack of Longitudinal Evaluation: Very few studies examined the longitudinal outcomes, such as how AI system performance or trust evolves over time in clinical workflows. This limits insights into the lifecycle of AI tools and their sustained value in real-world practice.

## 7. Conclusions

AI is transforming modern healthcare by enabling more accurate, efficient, and personalized medical services. This review explores AI in smart healthcare through two key perspectives: capability-based and functionality-based classifications. Currently, the field relies heavily on Narrow AI with limited memory, which supports tasks such as diagnostics, medical imaging, and predictive analytics within well-defined clinical workflows. As research advances, there is growing interest in AGI with Theory of Mind capabilities, aiming to develop systems that can understand context, interpret emotions, and support complex decision-making in areas like mental health and elderly care. While Superintelligent AI self-aware systems that surpass human intelligence remains theoretical, it raises critical ethical, regulatory, and philosophical questions. The continued development and integration of AI in healthcare demand close collaboration among clinicians, technologists, ethicists, and policymakers to ensure these systems are ethical, interpretable, and equitable. Ultimately, the goal is not to replace human clinicians but to enhance decision-making, reduce system burdens, and improve patient outcomes across diverse populations.

## Figures and Tables

**Table 1:** Comparative analysis of existing reviews vs. this work.

Study (Year)	Scope	Focus Area	Framework Type	Advantages	Limitations
[10]	General AI in Healthcare	Deep learning	Application-based	Broad overview of DL in diagnostics	No functional or capability-based categorization
[38]	XAI in Healthcare	Interpretability	Technical taxonomy	Introduced interpretability challenges in clinical AI	No classification of AI types or deployment stages
[23]	AI for Radiology	Diagnostic imaging	Narrow AI case study	Strong benchmarking of imaging models	Specific to radiology; lacks generalizability
[31]	Personalized Medicine	Predictive modeling	Limited memory-based	Personalized care pathway insights	Focused on narrow, reactive AI only
[34]	Conversational AI	Mental health bots	Emotional modeling	Emphasized empathy-aware dialogue systems	Does not generalize to other AI functionalities
[39]	Trust in Medical AI	Regulatory	Human-centered AI design	Excellent coverage of XAI + uncertainty estimation	Missing systematic tech-to-function mapping
This Work (2025)	Smart Healthcare Systems	AI Capabilities and Functionalities	Dual Framework (Capability + Functionality)	Holistic synthesis, new classification, tech-function alignment	Real-world deployment data are limited

**Table 2:** Study selection summary.

Selection Stage	Number of Records
Initial search hits	800
Duplicates removed	278
Title/abstract screened	522
Full-text articles reviewed	148
Final studies included that mainly focused on this topic	42

**Table 3:** Data extraction items for capability and functionality mapping.

Variable	Description
Study Information	Authors, year, country, journal
AI Capability Type	Narrow AI, General AI (AGI), Superintelligent AI
AI Functional Type	Reactive, Limited Memory, Theory of Mind, Self-Aware
Clinical Use Case	Diagnosis, triage, prognosis, robotic surgery, mental health, etc.
AI Technique	CNN, RNN, LLM, transformer, federated learning, etc.
Data Type Used	Imaging, EHR, genomic data, audio/textual data
Deployment Setting	Simulated lab, hospital-based, telemedicine, wearable device
Outcome Focus	Accuracy, interpretability, empathy, adaptability, autonomy

**Table 4:** Capability-based classification of AI in smart healthcare.

Capability Level	Core Trait	Current Use	Cognitive Scope	Clinical Role	Representative Systems
Narrow AI	Task-specific learning	Diagnostic imaging, chatbots, EHR prediction models	Limited to trained tasks	Assistive tools	DeepMind, Zebra, Aidoc, Wysa
General AI (AGI)	Cross-domain reasoning	Multimodal modeling, adaptive LLMs	Context-aware, human-like	Augmented clinician	Med-PaLM, GatorTron
Superintelligent AI	Surpasses human cognition	Theoretical	Beyond human capacity	Autonomous healthcare leader	Not yet realized

**Table 5:** Functionality-based classification of AI in smart healthcare.

Functional Type	Core Behavior	Healthcare Applications	Memory or Learning
Reactive Machines	Respond to present inputs only	ICU alerts, rule-based diagnostics, infusion control	No memory
Limited Memory Systems	Learn from historical data, no continuous learning	Imaging analysis, EHR-based risk prediction, wearable monitoring	Short-term memory
Theory of Mind	Understand user emotions and intentions	Empathy-aware chatbots, geriatric AI, adaptive clinical communication	Emotion/context modeling
Self-Aware AI	Model internal state and confidence	XAI, uncertainty-aware systems, adaptive therapeutic agents	Meta-cognition (early features)

**Table 6:** Integrative perspectives of AI capabilities and functionalities in smart healthcare.

Perspective	Current Use in Smart Healthcare	Functional Description
Narrow AI + Limited Memory	Clinical decision support, imaging, diagnostics, mental health bots	Uses historical data to make task specific decisions; no real-time learning or cross-domain flexibility
AGI + Theory of Mind	Early stage LLMs, emotion-aware chatbots, adaptive clinical assistants	Attempts human like reasoning and emotion modeling using multimodal, contextual data; not yet fully realized
Superintelligent AI + Self-Awareness	Theoretical; explored in XAI and ethical AI research	Hypothetical systems with full autonomy, self reflection, and ethical cognition; no clinical deployment

**Table 7:** Challenges and considerations in AI-driven smart healthcare.

Challenge	Description	Impact	Mitigation Strategy	Reference
Bias and Fairness	AI systems can reflect or amplify biases present in training data, affecting fairness across demographics.	Undermines trust and may lead to healthcare disparities.	Use diverse training data; implement fairness audits.	[124]
Interpretability	AI models, especially DL, often lack transparency, making it difficult for clinicians to trust outputs.	Limits clinical adoption and medico legal accountability.	Incorporate explainable AI (XAI) models and visualizations.	[38]
Regulation	AI deployment requires compliance with evolving legal and ethical frameworks.	Regulatory uncertainty slows innovation and deployment.	Develop adaptive, region-specific AI policies.	[125]
Data Security	Storing and sharing sensitive patient data raises concerns around privacy, encryption, and misuse.	Breaches may lead to legal liability and patient harm.	Employ federated learning and differential privacy.	[126]
Clinical Integration	Embedding AI into existing clinical workflows without disrupting care delivery is technically and culturally complex.	Causes resistance among staff and workflow inefficiency.	Co-design solutions with clinicians for smooth adoption.	[127]
Infrastructure and Cost	High development, deployment, and maintenance costs limit access in resource-constrained healthcare settings.	Restricts scalability and global AI implementation.	Invest in cloud infrastructure and public and private partnerships.	[128]
